# The Association Between Stress and Injury: A Prospective Cohort Study Among 186 First-Year Contemporary Dance Students

**DOI:** 10.3389/fpsyg.2021.770494

**Published:** 2021-11-05

**Authors:** Diana van Winden, Rogier M. van Rijn, Geert J. P. Savelsbergh, Raôul R. D. Oudejans, Janine H. Stubbe

**Affiliations:** ^1^Codarts Rotterdam, University of the Arts, Rotterdam, Netherlands; ^2^Department of Human Movement Sciences, Vrije Universiteit Amsterdam, Amsterdam Movement Sciences, Amsterdam, Netherlands; ^3^PErforming Artist and Athlete Research Lab (PEARL), Rotterdam, Netherlands; ^4^Institute of Brain and Behavior, Amsterdam, Netherlands; ^5^Faculty of Sports and Nutrition, Amsterdam University of Applied Sciences, Amsterdam, Netherlands; ^6^Rotterdam Arts and Sciences Lab (RASL), Rotterdam, Netherlands; ^7^Department of General Practice, Erasmus University Medical Center, Rotterdam, Netherlands

**Keywords:** pre-professional, psychological, stress, injury, workload, stressors, dance, performing arts

## Abstract

The demanding environment that contemporary dance students are exposed to could result in high stress levels, which can influence injury susceptibility. Therefore, this study aims to investigate the association between stress and injuries. In the period between September 2016 and March 2020, four cohorts of first-year dance students (*N* = 186; mean age 19.21 ± 1.35 years) were followed for one academic year. Each month, general stress was assessed on a 0–100 visual analogous scale. The Oslo Sports Trauma Research Center Questionnaire on Health Problems was used on a monthly basis to monitor injuries. Injuries were defined as “all injuries” (i.e., any physical complaint irrespective of the need for medical attention or time-loss from dance) and “substantial injuries” (i.e., leading to moderate/severe/complete reductions in training volume or performance). Mann–Whitney tests were performed to measure differences in general stress levels between injured and injury-free students, while repeated-measures ANOVA were performed to investigate whether general stress scores increased before and during injury occurrence. The overall average monthly general stress score over all cohorts for all students was 39.81. The monthly general stress scores ranged from 31.75 to 49.16. Overall, injured and substantially injured students reported higher stress scores than injury-free students, with significant differences in 3 out of the 9 months for all injuries (September, October, March, *p* < 0.05), and in 5 months for substantial injuries (September, October, November, December, April, *p* < 0.05). Within the 3-month period before and during injury occurrence, a (marginally) significant linear effect of general stress across the time periods was found for all injuries [*F*(1.87,216.49) = 3.10, *p* = 0.051] and substantial injuries [*F*(2,138) = 4.16, *p* = 0.018]. The results indicate an association between general stress and injuries. Future research should focus on effects of varying stress levels on injury risk using higher sampling frequency, for instance by measuring weekly since stress levels are likely to fluctuate daily. Practically, strategies aiming at stress reduction might have the potential to reduce the burden of dance injuries and may have positive outcomes for dancers, teachers, schools, and companies.

## Introduction

Contemporary dance includes a variety of styles and genres of dance and continues to grow in popularity (Martin, 2013). It mainly represents a fusion of styles and has specific aesthetic values; choreographers classify common traits such as experimental elements, a conceptual framework, or the inclusion of improvisation, text, or multimedia elements (Giguere, 2018). Contemporary dance training requires advanced physical and artistic skills and may consequently increase dancers’ susceptibility to injury (Kenny et al., 2016). Reported injury incidence rates among pre-professional dancers, ranging from 0.77 to 4.71 injuries per 1,000 h of dance, have indicated that dance is a high-risk activity (Kenny et al., 2016; van Winden et al., 2019).

Physiological and biomechanical risk factors for injuries have traditionally been dominant within the history of dance research (Mainwaring and Finney, 2017). Only a few studies have focused on the mental risk factors of injuries in dance students, although psychological skills can be considered modifiable (Kenny et al., 2016) and might affect the occurrence of dance injuries (Mainwaring and Finney, 2017). For instance, studies including pre-professional dance students demonstrated associations between injuries and psychological coping skills (Noh et al., 2005, 2007) and perceptions of fatigue (Liederbach and Compagno, 2001).

In order to provide a theoretical framework to clarify the relationship between mental aspects and (sports) injury occurrence, Williams and Andersen (1998) proposed the stress-and-injury model, which is supported by a more recent meta-analysis (Ivarsson et al., 2017). The meta-analysis showed that a strong stress responsivity has the strongest association with injury risk in sports compared to the other variables that are connected to injury risk according to the model (e.g., personality, history of stressors, coping). The model suggests that the stress response in a potentially stressful situation can influence injury risk due to increased muscle tension and decreased coordination, a lack of focus and increased self-consciousness (Williams and Andersen, 1998; Staufenbiel et al., 2013; Ivarsson et al., 2017). Moreover, prolonged stress can cause changes in brain functions that increase the risk of poor decision-making (Ivarsson et al., 2017), which has also been related to increased injury risk (Gabbett et al., 2012). The model can be applied to dance-specific examples according to a literature review of Pollitt and Hutt (2021), as for instance done by van Winden et al. (2020b). In line with the stress-and-injury model, the results of the systematic review of Mainwaring and Finney (2017) also showed that stress, psychological distress, coping and personality were associated with risk of dance injury.

Stress occurs when the environmental demands exceed the abilities of an individual to cope with the demands of specific events or experiences (e.g., stressors) (Lazarus and Folkman, 1984). These stressors can arise from both the dancer themselves and the dance environment, such as: high expectations from others, competitive auditions, and a demanding training schedule (Krasnow et al., 1999; Adam et al., 2004). In line with these perceived stressors, the most reported types of mental health issues among contemporary dance students were general anxiety, stress due to external factors and constant tiredness (van Winden et al., 2020a).

Studies including pre-professional ballet dancers have indicated associations between injuries and general negative stress (Mainwaring et al., 1993), as well as dance-specific negative stress (Mainwaring et al., 1993; Krasnow et al., 1999). Furthermore, stress has been associated with injury-caused absence from dance activities and prolonged injury duration in (pre-)professional ballet dancers (Adam et al., 2004; Noh et al., 2005). However, the current body of research focusing on stress and dance injuries is insufficient to draw decisive conclusions (Kenny et al., 2016; Mainwaring and Finney, 2017). Prospective cohort studies are needed (Kenny et al., 2016), as longitudinal and frequent monitoring of perceived stress may provide further insight into the changes in stress levels in relation to the dynamic nature of injury occurrence (van der Does et al., 2017).

In addition, most of the previously mentioned studies did not include (contemporary) dance students. Especially within the academic setting, injuries can have a tremendous effect leading to hindered artistic development due to absences from dance activities (Kenny et al., 2016), and even study delay or early drop-out of university. Furthermore it is possible that the specific academic environment could result in even higher stress levels compared to the general dance setting, due to, for example, the limited timeframe for achieving artistic and academic goals (Weigert, 2005). Besides, it is possible that all new (i.e., first-year) dance students are at an elevated level of injury risk, due to stressors such as moving, managing finances, and homesickness (Pollitt and Hutt, 2021). Therefore, a prospective cohort study with four cohorts of first-year contemporary dance students over the course of a full academic year was performed to gain more insight into the association between general stress levels and injuries (i.e., physical complaints irrespective of the need for medical attention or time-loss from dance). We hypothesize that students perceive more stress when injured.

## Materials and Methods

### Participants

Four cohorts of first-year contemporary dance students of Codarts Rotterdam, University of the Arts, Netherlands (*N* = 186), were prospectively followed during one academic year (September to June) from the study year 2016/2017 until 2019/2020. Due to the COVID-19 lockdown, results from March 2020 until June 2020 were excluded from the analysis, as students were not able to follow regular classes during that period. Students were enrolled in a 4-year educational program of either a Bachelor Dance or Bachelor Dance Teacher. Both Bachelor programs focus on acquiring the required technical dance competencies with classes containing a wide range of modern techniques (e.g., Cunningham, Graham, Laban, Limon), (modern) jazz, ballet, and “floorwork.” In addition, a large number of guest teachers contribute their personal styles, often inspired by their own development and research. Furthermore, performance creative skills (i.e., improvisation, composition, and drama) are important. Besides, health classes including basic knowledge of nutrition, anatomy and (sport)psychology are being offered within the first-year of the Bachelor Dance’s curriculum. Within the first-year of the Bachelor Dance Teacher, students have classes on reflection, communication and dealing with feedback.

### Procedures

Data was collected at regular intervals for management and educational purposes and data collection was embedded in the curriculum. All students were informed about the procedure and provided written consent in accordance with the Declaration of Helsinki. Ethical approval for the study was provided by the Medical Ethics Committee Erasmus MC of Rotterdam, Netherlands (MEC-2019-0163).

During the first month of each academic year, baseline characteristics were recorded including age (years), sex (male/female), BMI (kg/m^2^), educational program (Bachelor Dance or Dance Teacher), and 1-year history of injury (yes/no). One-year injury history was defined as “any physical complaint resulting in a fulltime loss of dance activities (e.g., participation in class, rehearsal, performance) for at least 1 week beyond the day of onset in the past year” (van Seters et al., 2020, p. 2). During the academic year, all students were asked to complete monthly questionnaires on their physical and mental health through the Performing artist and Athlete Health Monitor (PAHM). PAHM was developed by Codarts Rotterdam and is used to monitor physical and mental health in pre-professional and professional performing artists and athletes (Stubbe et al., 2018; Karreman et al., 2019; van Winden et al., 2019). This system consists of several questionnaires and items [e.g., visual analog scale (VAS) on pain; VAS stress; Oslo Sports Trauma Research Center Questionnaire on Health Problems; injury characteristics; items on sleep quality, feelings and emotions, satisfaction with rehearsals and performances].

### Stress Registration

A visual analog scale (VAS) was used to measure perceived general stress scores on a monthly basis. Students indicated their general stress scores on a scale ranging from 0 (no stress) to 100 (extreme amount of stress). The VAS is frequently used in stress assessment and several validity studies have highlighted its psychometric properties. The VAS is at least as sensitive as other stress scales (i.e., 14-items Perceived Stress Scale) (Lesage et al., 2012), is significantly correlated with objective stress measurements such as cardiovascular parameters (e.g., heart rate, blood pressure) (Hulsman et al., 2010), shows satisfactory reliability (Lesage et al., 2009), and inter-judge reliability (Lesage et al., 2011). No minimal clinically important difference has been determined (Rotter et al., 2020).

### Injury Registration

The Oslo Sports Trauma Research Center (OSTRC) Questionnaire on Health Problems is one component of the monthly questionnaire and consists of four key questions on the consequences of health problems on dance participation, training volume, performance and the degree to which students perceive any symptoms (Clarsen et al., 2014). Possible answers ranged from 0 (no problem, no reduction, no effect, or no symptoms) to 25 (cannot participate at all or severe symptoms) (Clarsen et al., 2013). Questions 1 and 4 were scored on a four-point scale (0, 8, 17, and 25), while questions 2 and 3 were scored on a five-point scale (0, 6, 13, 19, and 25). The OSTRC Questionnaire has a high internal consistency, with a Cronbach’s alpha of 0.96, good face validity (Clarsen et al., 2013, 2014), and has previously been used within the performing arts (van Seters et al., 2020; Kenny et al., 2018; Stubbe et al., 2018, 2021; van Winden et al., 2019, 2020a,b).

The severity of a health problem was calculated by the sum score of the four questions (scale 0–100) according to the method proposed by Clarsen et al. (2013). If the severity score was higher than zero, a health problem was registered and the student was asked whether the health problem was an injury, mental complaint, or other problem. An injury was defined as “any physical complaint sustained by a dancer resulting in a severity score higher than zero (i.e., leading to consequences on participation, training volume, and/or performance), irrespective of the need for medical attention or time-loss from dance activities” (van Winden et al., 2019, p. 2). Students were categorized as having a substantial injury if they reported problems leading to moderate or severe reductions in training volume (value ≥13 on question 2 of the OSTRC Questionnaire) or moderate, severe, or complete reductions in performance (value ≥13 on question 3 of the OSTRC Questionnaire) (Clarsen et al., 2014).

### Statistical Analyses

Statistical analyses were performed using SPSS Version 26 (IBM Corp., Armonk, NY, United States) and statistical significance level was set at an alpha level >0.05. Missing data were excluded pairwise. Descriptive statistics were used to describe baseline characteristics and general stress scores using medians and ranges or frequency and proportions (%). Body mass index was calculated from dancers’ baseline height and weight. Non-parametric tests were used as not all of the data had a normal distribution as indicated by the Shapiro–Wilk tests of Normality. Mann–Whitney tests for each month were used to compare general stress scores between injured and injury-free students, and between substantially injured and non-substantially injured students. Effect sizes (*r*) were measured by dividing the z-score with the square root of *N* (size of the study sample) (Field, 2009).

**FIGURE 1:**
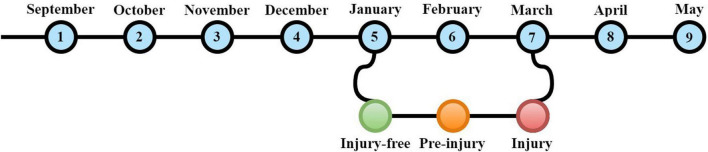
Timeline example with specific time periods marked as “injury-free period,” “pre-injury period,” or “injury period.”

According to the injury registration, specific “time periods” were marked as “injury-free period,” “pre-injury period,” or “injury period” (Figure 1), which is adjusted from the method previously used by van der Does et al. (2017). For example, if a student reported a substantial injury in March, January was marked as an injury-free period, February was classed as the pre-injury period, with March being the injury period. Repeated-measures ANOVA with follow-up polynomial contrasts and pairwise comparisons were performed to investigate whether general stress scores increased within this 3-month period. Only injured students were included in these analyses. For each injured student, only the first “complete set” of scores was used. Therefore, the months after the injury period were not taken into account.

## Results

### Participants

A total of 193 students from the Bachelor Dance and Bachelor Dance Teacher were prospectively followed during their first academic year. A total of 186 students (68.3% females) agreed to participate and were all included in the present study, with a median age of 18.94 (range 17.00–28.60) (Table 1). Within the study years 2016/2017, 2017/2018, and 2018/2019 there were 46 participants, whereas study year 2019/2020 included 48 participants. In total, 1,530 monthly questionnaires were sent to these students (maximum of 9 per student) and 1,391 were completed, resulting in a response rate of 90.9%.

**TABLE 1:** Baseline characteristics shown as median (range) or number (percentage).

	**Overall**
***N***	186
**Education program (Bachelor Dance)**	121 (65.1%)
**Sex (female)**	127 (68.3%)
**Age (years)**	18.94 (17.00–28.60)
**BMI (kg/m^2^)^#^**	20.81 (17.73–29.13)
One-year history of injury (yes)*	60 (32.3%)

*^#^Missing data of *N* = 1.*

*∗Missing data of *N* = 3.*

### Overall General Stress Scores

**FIGURE 2:**
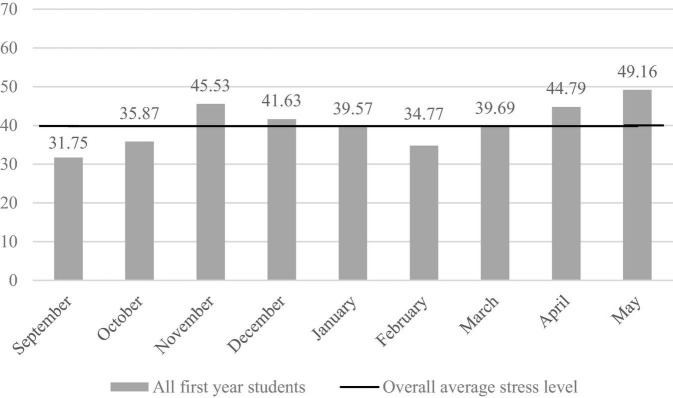
Stress levels throughout the academic year for all first-year students, including the overall average stress level.

The overall average monthly general stress score was 39.81 (SD = 23.24) over all included cohorts and for all first-year students. Figure 2 demonstrates that the monthly stress scores ranged from 31.75 to 49.16.

### Difference in General Stress Scores for Injured and Injury-Free Students

Differences in general stress scores for either injured (i.e., “all injuries”: students who indicated at least one physical complaint irrespective of the need for medical attention or time-loss from dance during the previous month) and injury-free students (i.e., students who did not indicated any physical complaints), or substantially injured (i.e., students who indicated at least one physical complaint leading to moderate/severe/complete reductions in training volume or performance) and non-substantially injured students (i.e., injury-free and non-substantially injured students) are shown in Table 2. During 3 out of the 9 months (i.e., September, October, and March), injured students reported significantly higher general stress scores than their injury-free peers, with small effect sizes. For the remaining 6 months, stress scores of injured students compared to injury-free students were higher across 5 of the 6 months, although not significantly. Furthermore, general stress scores were significantly higher in substantially injured students compared to non-substantially injured students for 5 out of the 9 months (i.e., September, October, November, December, and April), with small to medium effect sizes. For the remaining 4 months, stress scores of substantially injured students compared to non-substantially injured students were higher, although not significantly.

**TABLE 2:** Stress scores for injured and injury-free students, and substantially and non-substantially injured students.

	**September**	**October**	**November**	**December**	**January**	**February**	**March**	**April**	**May**
Mean injury-free	28.9	32.39	44.32	41.38	40.91	34.73	36.33	43.21	48.42
Mean injured	36.84	42.96	48.19	42.28	37.51	34.82	43.76	47.24	50.48
Significance level (*p*)	0.04*	0.002**	0.355	0.689	0.405	0.861	0.047*	0.259	0.70
Effect sizes (r)^#^	−0.16	−0.23	−0.07	−0.03	−0.06	−0.01	−0.18	−0.10	−0.04
Mean non-substantially injured	30.3	33.68	42.49	40.09	38.76	33.97	38.58	42.85	48.1
Mean substantial injured	44.17	54.72	63.92	53.42	43.89	39.78	45.83	56.33	54.58
Significance level (*p*)	0.038*	0.00**	0.00**	0.021*	0.273	0.273	0.217	0.017*	0.238
Effect sizes (r)^#^	−0.16	−0.30	−0.32	−0.18	−0.08	−0.08	−0.11	−0.21	−0.11

*^∗^Significance at *p* < 0.05; ^∗∗^significance at *p* < 0.001.*

*^#^Small effect size: *r* = 0.1–0.3; medium effect size: *r* = 0.3–0.5; large effect size: *r* ≥ 0.5 (Field, 2009).*

### General Stress Scores Before and During the Occurrence of an Injury

**FIGURE 3:**
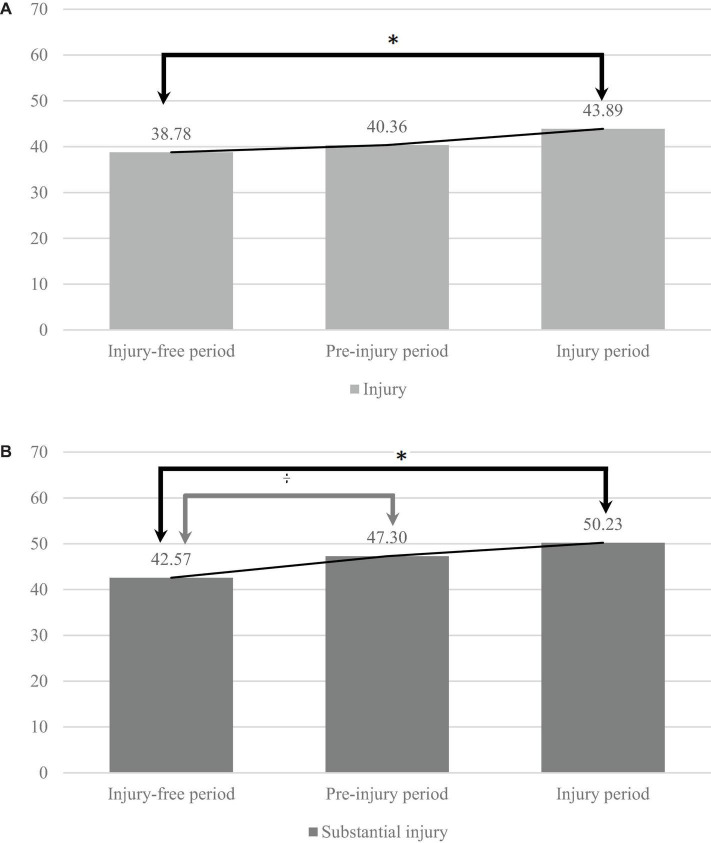
**(A,B)** Stress scores in the injury-free, pre-injury, and injury periods for all injuries and substantial injuries, with significant differences between the injury-free and injury periods for both injury definitions (*), as well as between the injury-free and pre-injury period for substantial injuries (†), *p* < 0.05.

For both outcome measures, all injuries (*N* = 117) and all substantial injuries (*N* = 70), general stress scores increased from the injury-free period to the injury period (Figure 3).

With regards to all injuries, the assumption of sphericity was violated by Mauchly’s Test of Sphericity [χ^2^(2) = 8.55, *p* < 0.05]. As a result, the Greenhouse–Geisser correction was used. There was a marginally significant effect of “time period” on general stress scores: *F*(1.87,216.49) = 3.10, *p* = 0.051. Follow-up polynomial contrasts indicated a significant linear effect of stress scores increasing across the time periods (i.e., injury-free, pre-injury, and injury period), *F*(1,116) = 4.95, *p* = 0.028. Pairwise comparisons indicated that stress scores were significantly higher during the injury period (*M* = 43.89, SD = 22.80) compared to the injury-free period (*M* = 38.78, SD = 21.76, *p* = 0.028), but not compared to the pre-injury period (*M* = 40.36, SD = 22.47, *p* = 0.106). Further, stress scores in the pre-injury period were not significantly higher compared to the injury-free period (*p* = 0.385).

With regards to substantial injuries, there was a significant effect of “time period” on general stress scores: *F*(2,138) = 4.16, *p* = 0.018. Follow-up polynomial contrasts indicated a significant linear effect of stress scores increasing across the time periods, *F*(1,69) = 6.89, *p* = 0.011. Pairwise comparisons indicated that stress scores were significantly higher for the substantial injury period (*M* = 50.23, SD = 23.01) compared to the injury-free period (*M* = 42.57, SD = 22.35, *p* = 0.011), but not compared to the pre-injury period (*M* = 47.30, SD = 21.98, *p* = 0.293). Although, stress scores in the pre-injury period were significantly higher compared to the injury-free period (*p* = 0.045).

## Discussion

This study investigated the association between general stress levels and injuries among first-year contemporary dance students. It was hypothesized that students perceive more stress when injured. Our results confirmed these hypotheses, indicating an association between stress levels and injuries, especially substantial injuries.

The monthly general stress levels showed peaks in November, April, and May, consistent with important periods such as exams and special project weeks, and low levels in September and February, consistent with the start of the semester. To our knowledge, no dance-specific literature has reported stress scores similar to our “VAS” measurement method. Therefore, we compared our results to other performing arts and general work populations using the same method. The overall average general stress score (*M* = 39.81, SD = 23.24) could indicate a moderate stress level when compared with work-related literature, which classifies scores below 30 as low stress and above 60 as high stress among female teachers (Ritvanen et al., 2006). Furthermore, Stubbe et al. (2021) showed an similar average general stress score of 40.38 during a 6-month period for performing arts students (including dance, circus, and music theater students), in which a small part of the data has some overlap with our data. Rotter et al. (2020) showed an average stress score of 51.7 (SD = 21.4) for professional adult musicians who suffered from chronic neck pain in line with our stress score when sustaining a substantial injury (*M* = 50.23, SD = 23.01).

An 1-year injury incidence proportion of 67.6% (*N* = 125) for all injuries (i.e., any physical complaint leading to consequences on participation, training volume, and/or performance, irrespective of the need for medical attention or time-loss from dance activities) and 43.2% (*N* = 80) for substantial injuries (i.e., leading to moderate or severe reductions in training volume or moderate, severe, or complete reductions in performance) was recently indicated by van Rijn and Stubbe (2021) within the same sample of first-year contemporary dance students. In general, our between-subject analyses showed that injured students and substantially injured students reported higher general stress scores than their injury-free or non-substantially injured peers across all months, except for January, in which injury-free students scored higher than injured students. These results were significant for all injuries during 3 out of the 9 months, and for substantial injuries during 5 out of the 9 months. The results for January might differ due to the fact that January is a relatively “easy” month within the academic calendar and was preceded by a holiday break. Furthermore, significantly higher stress scores were found in the months September and October for (substantially) injured students, consistent with the start of the first semester. Sustaining an injury in the beginning of their academic career could result in higher stress, since students are still adjusting to their new environment (e.g., the intensity of classes, social aspects, teachers’ reactions toward injuries et cetera). These stressors arise on top of the stressors all new (i.e., first-year) dance students experience (e.g., moving, managing finances, and homesickness) (Pollitt and Hutt, 2021). In addition, within-subject analyses showed that general stress scores significantly differed between the injury-free period and the injury period for injured students and substantially injured students.

The indicated association between stress levels and injuries is in agreement with previous research. Studies including pre-professional ballet dancers found associations between injuries and negative (general and/or dance-specific) stress (Krasnow et al., 1999; Adam et al., 2004; Noh et al., 2005). In the sports literature, the stress-and-injury model of Williams and Andersen (1998) highlights the association between stress and injuries. Major psychosocial stressors (i.e., events resulting in major changes in the life of those affected) (Junge, 2000) and minor or everyday events (e.g., health, workload, or social issues) (Lazarus and Folkman, 1984) can have an effect on stress and thereby increase injury risk. Contemporary dance students are, for instance, exposed to life events, such as managing finances, homesickness, competitive auditions, approaching exams and performances and high expectations from others which can lead to great amounts of stress (Mainwaring et al., 1993; Adam et al., 2004; Pollitt and Hutt, 2021).

Furthermore, in-depth descriptive measures into the 3-month period before and during the occurrence of either all injuries or substantial injuries showed a significant linear effect of general stress scores increasing across the time periods for all injuries and substantial injuries. For both outcome measures, the stress scores were significant higher during the injury period (i.e., the month of injury occurrence). Previous dance literature has shown conflicting results regarding stress levels before an injury occurs, with most studies reporting no association between work-related stress and injuries (Mainwaring and Finney, 2017). One exception, Patterson et al. (1998) indicated that negative life events, which resulted in elevated stress, were significant predictors of subsequent injuries within ballet dancers, especially when dancers experienced low social support. These outcomes showed parallels with our results.

### Practical Implications

Although the literature of dance medicine and science has traditionally focused on physical risk factors of injuries, our results indicate that psychological variables in general may be associated with the occurrence of dance injuries, in agreement with recent reviews within dance (Mainwaring and Finney, 2017; Pollitt and Hutt, 2021). Psychological training programs aimed at reducing stress levels might have the potential to reduce the burden of dance injuries and may have positive outcomes for dancers and those in their environment (e.g., dance schools, teachers, directors, and health professionals), as recommended by Ivarsson et al. (2017) in the context of sports. According to previous literature, a reduction in injury risk is likely when students are able to manage their stress levels better (Fawkner et al., 1999). For example, Noh et al. (2007) showed that within young ballet dancers, imagery, self-talk and relaxation techniques enhanced psychological coping skills and reduced injury frequency and duration. Besides, mindfulness can be an effective instrument to achieve a relaxed state of body and mind (Arvinen-Barrow and Walker, 2013), and potentially reduce stress and the subsequent physiological (e.g., relaxation) and attentional (e.g., mindfulness) changes (Ivarsson et al., 2017). A recent study among university dance students indicated that improvements in mindfulness may help students with numerous general and dance-specific demands (Blevins et al., 2021).

Considering a different approach, the management of workload (i.e., the amount and intensity of dance classes, rehearsals, and performances) and training environment (e.g., dance floors, mirror use, noise, amount of breaks) could also aid toward protecting dancers’ physical and mental health (Grove et al., 2013; Rice et al., 2016), thus reducing stress levels. To illustrate, the monthly stress levels peaked in periods with exams and special project weeks. Moreover, dancers typically face an increase in demands prior to important performances, in contrast to sports where tapering periods are common before an important match (Balk et al., 2018). Lowering workload during stressful periods, as well as facilitating possibilities for dancers to leave their dance bubble (i.e., the social, professional and education environment that is connected to dance) for “mental detachment” by, for instance, taking time off after stressful periods or organizing non-dance activities (Balk et al., 2018), might help balance stress levels throughout the year, and thereby influence the injury burden. However, future research should look more closely at changes in training load on individual level preceding the onset of injury (Drew, 2015; Lee et al., 2017), in order to gain more insight into the stress-workload relationship.

### Strengths, Limitations and Recommendations

To our knowledge, this is the first study to gain insight into general stress levels over time and the association with injuries within first-year contemporary dance students. A major strength of the current study is the prospective design in which we used a monthly follow-up, resulting in low interference of recall bias for injuries. In addition, the response rate to the monthly questionnaire was very high (90.9%), as a result of integrating the online monitoring system and feedback tool with visual information about their personal data (i.e., PAHM) within the educational program (Richardson et al., 2017; van Winden et al., 2019).

Yet, there are a number of limitations to the present study. First, the used self-reported outcomes for injuries and general stress scores resulted in subjective data and limited diagnostic information. Therefore, in future research, we recommend to measure objective stress levels to supplement the subjective data, using physical parameters such as cortisol or amylase (Takai et al., 2004).

Second, students indicated an overall monthly general stress score, since administering questionnaires on a monthly basis yields a more practical health monitoring tool with higher compliance compared to, for instance, weekly (Richardson et al., 2017) or daily monitoring. However, stress levels are likely to fluctuate on a day-to-day basis (von Rosen et al., 2017a), whereby sudden changes and short periods of high stress could potentially be overlooked when using a monthly measuring method (van der Does et al., 2017). Therefore, future research should consider including higher sampling frequencies, for instance on weekly basis. Moreover, in line with the dynamic nature of stress, future research should examine the effects of intra-individual varying stress levels on injury risk. However, higher sampling frequencies are needed to adequately perform these in-depth analyses. To illustrate, a study among athletes indicated a significant increase in stress scores in the week prior to injury (Fawkner et al., 1999), showing a much shorter time period between changes in stress levels and injury occurrence than measured in our study (week versus month). Gaining insight in these intra-individual varying stress levels can, for instance, be achieved by using more sophisticated statistical models, such as joint models, in which survival and longitudinal sub-models are linked and specific, individual predictions can be included (Long and Mills, 2018).

Besides, the current study focused on students’ perceived general stress before or during the month of incurring an injury. Previous research has shown that perceived stress and recovery during the injury period differed between injured and healthy athletes; injured athletes perceive more stress and feel less recovery during the period that they were injured (Evans et al., 2012). Consequently, in order to inform return-to-dance interventions, it is essential to conduct further research into perceived stress and recovery levels during injury periods (van der Does et al., 2017). Furthermore, future research should include other mental and physical risk factors as well, especially due to the small to medium effect sizes found in this study. For instance, studies have shown that dance exposure (i.e., years of training, exposure hours) or poor aerobic capacity could be associated with injury risk (Kenny et al., 2016). In addition, studies on sports have indicated mental detachment (Balk et al., 2019), perceived recovery (van der Does et al., 2017), sleep volume (von Rosen et al., 2017a, b) and self-esteem (von Rosen et al., 2017a) as potential injury risk factors. Finally, the present study was conducted among a first-year contemporary dance student population. These results may help direct injury prevention in second-year students in light of the similarity across their curriculums. However, generalizability to third-year (and fourth-year) dance students is limited, as these curriculums differ to a larger extent. Besides, it is questionable if the results can be generalized to other populations such as pre-professional ballet dancers or professional dancers. Future directions in dance research should focus on large prospective cohort studies. These studies will allow us to perform subgroup analyses based on different dance populations.

## Conclusion

Overall, our results indicate an association between general stress levels and injuries in first-year contemporary dance students, in line with the stress-and-injury-model of Williams and Andersen (1998). Between-subject analysis indicated that injured and substantially injured students reported higher general stress scores than injury-free or non-substantially injured students, although not all monthly scores were significantly higher for injured or substantially injured students. Furthermore, within the 3-month period before and during injury occurrence, a significant linear effect of general stress scores increasing across the time periods was found for all injuries and substantial injuries, with the highest scores in the injury period. However, more research, especially into the effect of varying stress levels on injury risk, with higher frequencies (for instance with weekly measurements) is needed. For now, better management of workload and a focus on the possible effects of dance students’ stress levels, by including psychological training programs aimed at reducing stress levels, for instance based on mindfulness, might have the potential to reduce the burden of dance injuries and may have positive outcomes for dancers, teachers, schools and companies.

## Data Availability Statement

The raw data supporting the conclusions of this article will be made available by the authors, without undue reservation.

## Ethics Statement

The studies involving human participants were reviewed and approved by the Medical Ethics Committee Erasmus MC of Rotterdam, Netherlands (MEC-2019-0163). The patients/participants provided their written informed consent to participate in this study.

## Author Contributions

DW performed the data collection, analyzed the data, and wrote the manuscript. RR assisted with performing the data collection and analyzing the data. RO, GS, and JS initiated the study and contributed to the content of the article. All authors contributed to manuscript revision, read and approved the submitted version.

## Conflict of Interest

The authors declare that the research was conducted in the absence of any commercial or financial relationships that could be construed as a potential conflict of interest.

## Publisher’s Note

All claims expressed in this article are solely those of the authors and do not necessarily represent those of their affiliated organizations, or those of the publisher, the editors and the reviewers. Any product that may be evaluated in this article, or claim that may be made by its manufacturer, is not guaranteed or endorsed by the publisher.
